# An Unusual Terrible Triad Variant Associated with an Essex-Lopresti Injury

**DOI:** 10.1155/2021/8522303

**Published:** 2021-09-16

**Authors:** Luis M. Salazar, Abdullah Ghali, Jose M. Gutierrez-Naranjo, Thomas L. Hand, Anil K. Dutta

**Affiliations:** Department of Orthopaedics, UT Health San Antonio, San Antonio, TX, USA

## Abstract

Essex-Lopresti injuries and terrible triad injuries of the elbow are rare injuries that typically result from high-energy trauma such as falling from a height or a motor vehicle collision. However, the combination of an Essex-Lopresti injury and terrible triad injury is unique and poses a significant challenge for treatment as these injuries are independently associated with poor functional outcomes if they are not acutely diagnosed. We describe a case of a 19-year-old who presented with an unusual variant of a terrible triad injury associated with an Essex-Lopresti injury. The patient had a distal radioulnar joint (DRUJ) and elbow dislocation, a radial head and coronoid process fracture, and a distal radius fracture. Almost a reverse Essex-Lopresti, this injury was successfully managed with open reduction and repair of the distal radius, radial head, and damaged ligaments in the elbow, along with an internal joint stabilizer (IJS).

## 1. Introduction

The Essex-Lopresti injury (ELI) is exceedingly rare and accounts for about 1% of all radial head fractures [1]. These injuries are characterized by a combination of 3 factors: distal radioulnar joint dislocation, a radial head fracture, and a rupture of the interosseous membrane (IOM). Although first reported by Brockman in 1930 and Curr in 1946, this fracture was formally named in 1951 after the British orthopaedic surgeon Peter Essex Lopresti described 2 cases [2–4]. Usually stemming from high-energy trauma, these injuries are caused by longitudinal force applied to the radius. If the DRUJ dislocation and IOM rupture are not recognized in the acute setting, these injuries can result in chronic complications such as wrist pain, subluxation, and further translation of the radius [4, 5]. Even more rare are variants of Essex-Lopresti injuries (ELIs) associated with a terrible triad in the ipsilateral elbow [6]. A terrible triad injury is composed of an elbow dislocation, a radial head or neck fracture, and a coronoid fracture. Similar to ELIs, ideal management remains controversial as there are a lack of studies to solidify the best surgical management [7]. We present a case of terrible triad variant associated with an Essex-Lopresti and distal radius fracture in a 19-year-old. We hope that our experience will highlight important treatment options and shed light on this infrequent presentation.

## 2. Case Presentation

**Figure 1:**
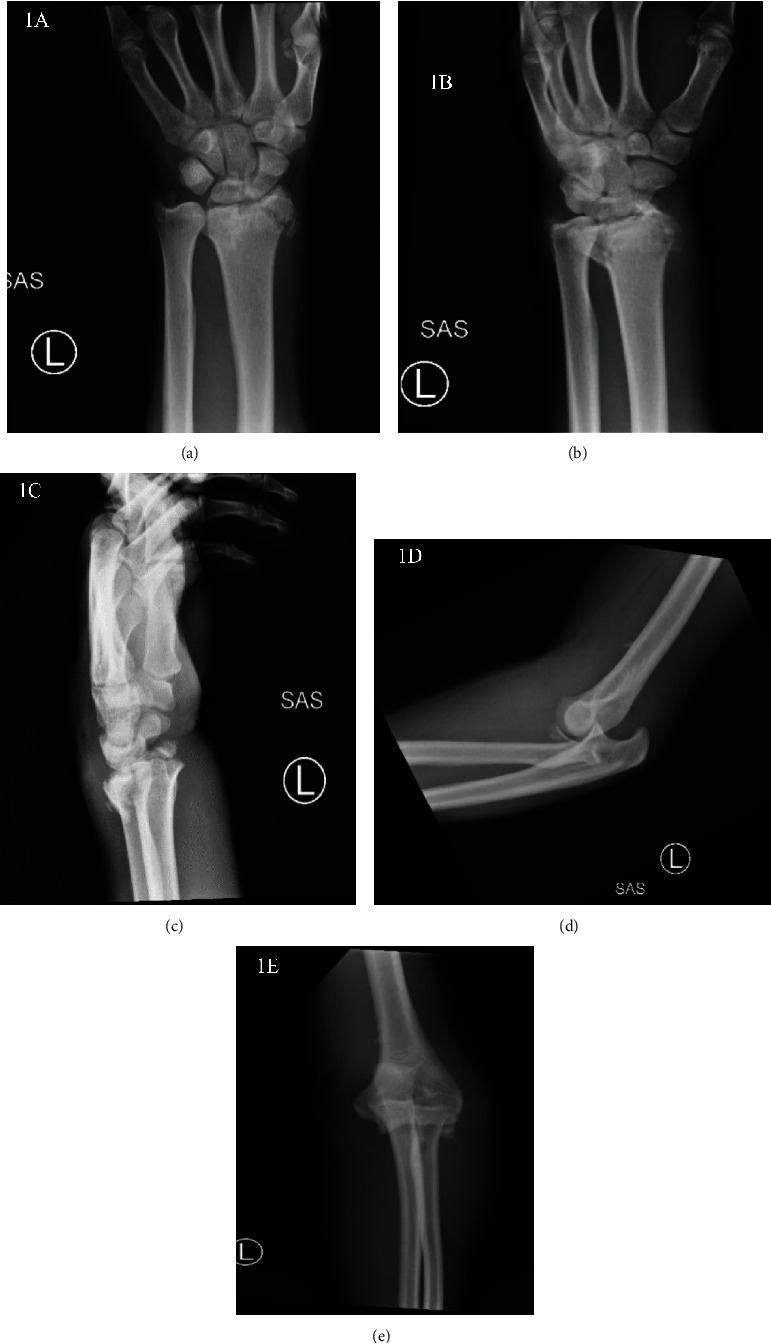
Left wrist PA (a), oblique (b), and lateral (c) injury radiographs along with left elbow lateral (d) and AP (e) injury radiographs. Left elbow posterior dislocation with fracture fragment along the anterolateral aspect of the elbow. Comminuted intraarticular left distal radius fracture.

A 19-year-old male fell onto his left wrist and left elbow while working on a roof, reporting immediate pain and swelling in his left wrist and elbow. On examination, there was significant soft tissue swelling with an obvious deformity in the left wrist and left elbow; skin and neurovascular functions were intact. Initial radiographs requested in the emergency department showed a comminuted intraarticular left distal radius fracture with mild dorsal angulation (Figures 1(a)–1(c)) and a posterior dislocation of the left elbow with a displaced fracture fragment along the anterolateral aspect of the elbow (Figures 1(d) and 1(e)). In addition, there was a mildly displaced ulnar styloid fracture and nondisplaced scaphoid wrist fracture. Subsequently, a CT and an MRI was requested, showing evidence of a coronoid process fracture as well as high-grade to complete tears of the lateral ulnar collateral and radial collateral ligaments. High-grade partial-thickness tears of the common flexor tendon were also seen. Due to the severity of the injury, the radiologist report could not rule out injury to the interosseous membrane and required clinical correlation. The elbow was reduced with the assistance of a C-arm followed by a closed reduction of the left wrist. The left upper extremity was stabilized with a double sugar tong splint with IO molding and ulnar deviation prior to the surgical fixation.

**Figure 2:**
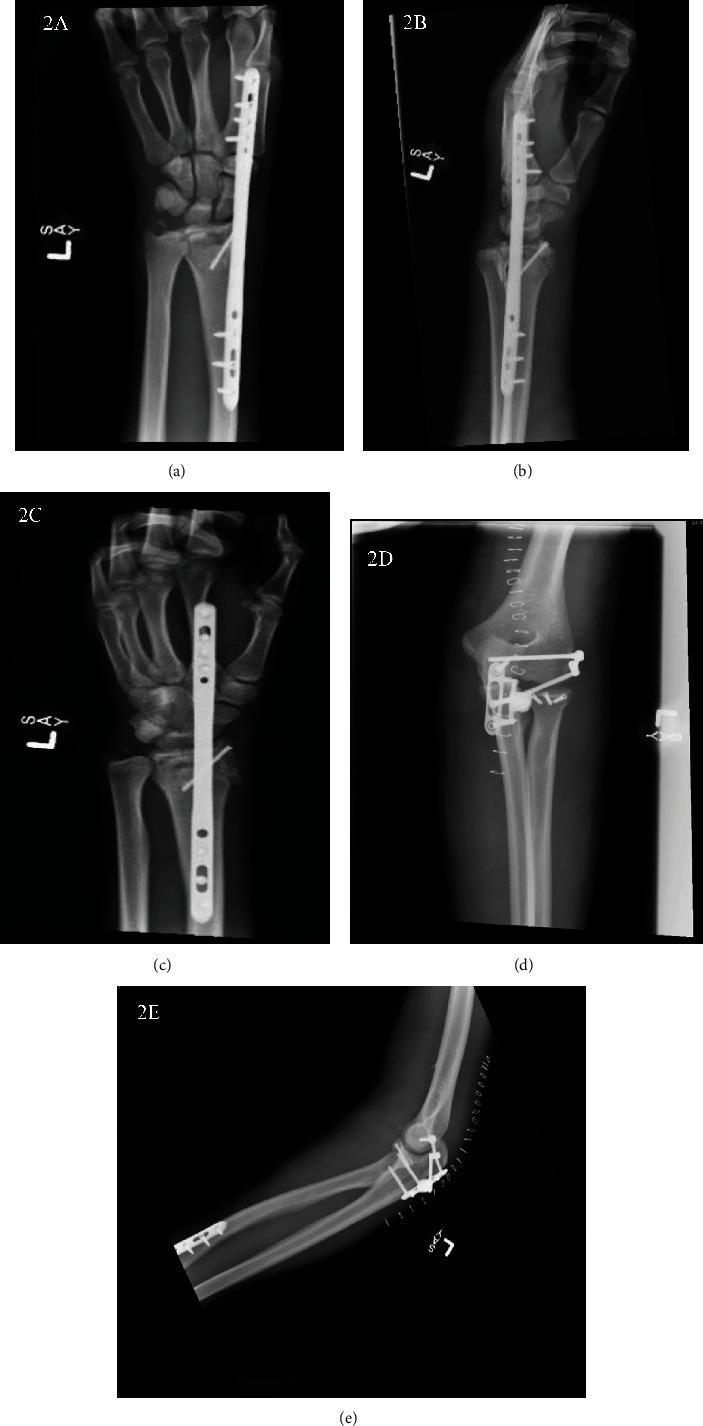
Postoperative radiographs of the plate fixation in the left wrist PA (a), lateral rotation (b), and external rotation (c) along with postoperative AP (d) and lateral radiographs (e) of the left elbow showing IJS placement.

Surgical treatment was managed on the third day of admission. A dorsal spanning plate was used to reduce the distal radius (Figures 2(a)–2(c)). A Kaplan approach was used to perform an arthrotomy on the unstable elbow and reduce the radial head. Intraoperatively, hypermobility at the level of the distal radioulnar joint (DRUJ) and axial and coronal instability of the forearm was observed, confirming intraosseous membrane damage and an Essex-Lopresti injury [8, 9]. The lateral collateral ligament (LCL) was repaired, and a left internal joint stabilizer (IJS) was placed (Skeletal Dynamics, Miami, FL) (Figures 2(d) and 2(e)). Good concentric flexion and extension of the elbow and radial head were achieved. A posterior splint was placed in the elbow with the wrist in neutral position to protect from any instability with passive range of motion being allowed.

**Figure 3:**
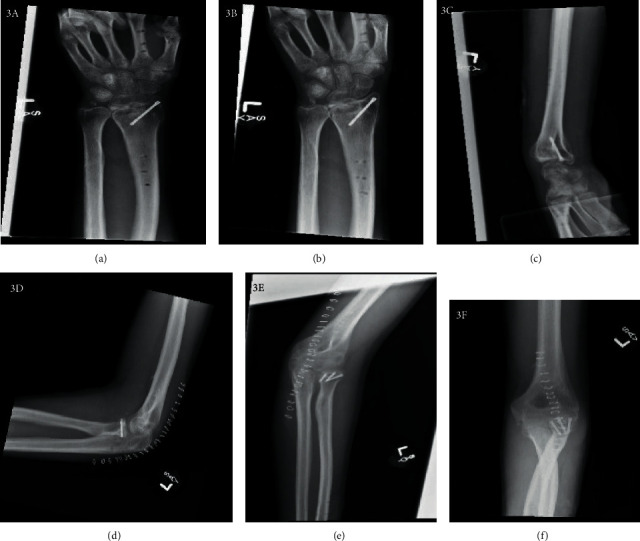
Radiographs at final follow-up showing healed fractures. Left wrist PA (a), external rotation (a) and lateral rotation (c). Left elbow lateral (d) and AP 2 views (e, f).

At 6 weeks of follow-up, the patient presented to the clinic with minimal pain but diminished ROM about the elbow, forearm, wrist, and hand. He was able to flex and extend about the elbow from 30 to 90 degrees, pronate-supinate to 45 and 45 degrees, and there was limited flexion/extension about the thumb, index, and middle fingers. At 3 months, the spanning plate of the left wrist was removed and tenolysis of the extensor pollicis longus was performed due to adherence to the dorsal spanning plate (Figures 3(a)–3(c)). Tenolysis of the adjacent extensors, including the digital extensors and wrist extensors, was also done, increasing flexion and extension of the wrist to 30 degrees in both planes. The IJS and dorsal base plate of the left elbow were removed, proceeding with an arthrotomy of the radiocapitellar joint to improve extension from 20 degrees to full extension (Figures 3(d)–3(f)). Flexion also improved from 120 to 125 degrees.

## 3. Discussion

Fracture-dislocations of the forearm, such as Galeazzi or Monteggia injuries, are well described in the literature. ELIs are a complex, third fracture-dislocation type that is rare and made up of a DRUJ dislocation, IOM rupture, and a radial head fracture. Variants of an ELI coupled with another fracture-dislocation in the elbow are sparsely reported in the literature. More specifically, an ELI and a terrible triad in the elbow, including an elbow dislocation and a radial head and coronoid process fracture, has only been reported once in the literature [6]. Also, one case of reverse ELI with anteromedial radial head dislocation, DRUJ dislocation, and distal radius fracture has been reported [10]. To the best of our knowledge, we present the first documented case of an ELI, a terrible triad, and a distal radius fracture. Our case report highlights the infrequency of this presentation as well as the treatment challenge that this variant represents.

Making up less than 1% of all radial head fractures, ELIs are often overlooked and difficult to diagnose [1]. In their series on 20 patients with this injury, Trousdale et al. showed that only 25% of patients were correctly diagnosed initially [11]. ELIs are underdiagnosed because attention is typically directed towards the radial head fracture [12–14]. Patients present with subtle wrist symptoms and frequently lack pain or swelling in the wrist or forearm [4]. Clues to the diagnosis include exaggerated prominence of the distal ulna, pain, and instability about the DRUJ and a lack of ulnar deviation on pronation-supination of the forearm [15]. Historically, missing the DRUJ dislocation and IOM disruption in the acute setting has led to poor clinical outcomes and impaired function [11]. IOM rupture allows the radius to migrate proximally and results in devastating sequelae such as severe chronic wrist pain and weakness in the forearm and wrist [16, 17]. Therefore, it is imperative to perform a thorough clinical and radiologic exam of the wrist in patients presenting with a radial head fracture in order to rule out this uncommon presentation. Similarly, complex elbow dislocations such as terrible triad injuries pose a significant risk to chronic disability. Common complications include chronic elbow instability, arthrosis, nonunion or malunion, elbow contracture, ulnar neuropathy, and proximal radioulnar synostosis [18]. Moreover, surgical treatment of terrible triad injuries carries a high-complication rate with an average risk of 22% for reoperation [19].

ELIs generally occur from high-energy trauma such as motor vehicle collisions or falls from a height as in our patient. The mechanisms of an ELI involve disruptions of forearm stability and biomechanics. The ELI most commonly involves an axial load to a proximal forearm in pronation [1]. The primary stabilizer is the radial head at the proximal radioulnar joint (PRUJ). The main resistance to proximalization of the radial head is a proper articulation between the radial head and the capitulum. Secondary stabilizers are the DRUJ and the IOM [20]. The IOM allows load shifting from the radius to the ulna. Birkbeck et al. demonstrate no load shifts from the radius to the ulna in cadaver studies with dissected IOM and a triangular fibrocartilage complex (TFCC) [21]. Therefore, when a high-energy longitudinal force is applied to the wrist, these stabilizers are disrupted and the radius can translate proximally, resulting in impaction of the radial head [22]. Such an injury in conjunction with a terrible triad in the elbow and a distal radius fracture has not been described before in the literature.

Understanding the mechanisms at play in ELIs is important for management. Acutely, focusing on stabilizing the DRUJ with reduction and Kirschner wires leads to optimal outcomes [10, 17, 23]. Furthermore, emphasis on preserving the radial head has been well-established due to the devastating sequelae of further proximalization of the radial head [4, 23, 24]. Chronic forearm instability is common in radial head resections without replacement, so these should always be avoided [11, 14]. When the radial head is not salvageable, radial head replacement is recommended to mitigate further translation of the radius [13, 25, 26]. Our patient's injury presented a unique challenge because we needed to consolidate the principles of managing ELIs, terrible triads, and distal radius fractures. In the elbow, we proceeded with internal fixation of the radial head with a repair of the LCL and placement of an IJS. This preserved the radial head along with providing adequate stability for the terrible triad with an IJS [27, 28]. Distally, we reduced the radius with a dorsal spanning plate and achieved satisfactory support in the forearm without needing to reconstruct the IOM with a graft [16, 17].

## 4. Conclusion

We present the first documented case of a patient with an ELI, terrible triad, and distal radius fracture. Clinicians must examine patients thoroughly and have a high index of suspicion in any patient with a radial head fracture to prevent the serious complications associated with ELIs. Management of these variants is challenging, and we hope to contribute to the overall knowledge of these variants and offer a successful treatment strategy.
